# Golden Rules of Diagnosis and Treatment of Diseases

**Published:** 1911-04

**Authors:** 


GOLDEN RULES OF DIAGNOSIS AND TREATMENT OF DISEASES.—Aphorisms, Observations and Precepts on the Method of Examination and Diagnosis of Diseases, with Practical Rules for Proper Remedial Procedure. By Henry A. Cables, B. S., M. D., Professor of Medicine and Clinical Medicine, College Physicians and Surgeons, St. Louis, etc. St. Louis. 1911. C. V. Mosby Company. Cloth. 278 pages. Price, not stated.

This is really a very important and valuable book. Diagnosis is the most important part of the practice of medicine. Given the diagnosis, any text-book will give you the treatment. It is notorious that not sufficient attention is given it in most medical schools. It stands in relation to practice about as does Mrs. Hale's injunction in her famous recipe "How to Cook a Hare"—"first, get your hare," does to the cooking. The "Red Back" recommends Dr. Cables' little book to its readers as eminently "worth while."—D.

